# Correction: Using an RNA aptamer probe for super-resolution imaging of native EGFR

**DOI:** 10.1039/c8na90005a

**Published:** 2019-01-08

**Authors:** Qiuyan Yan, Mingjun Cai, Lulu Zhou, Haijiao Xu, Yan Shi, Jiayin Sun, Junguang Jiang, Jing Gao, Hongda Wang

**Affiliations:** State Key Laboratory of Electroanalytical Chemistry, Research Center of Biomembranomics, Changchun Institute of Applied Chemistry, Chinese Academy of Sciences Changchun Jilin 130022 P. R. China hdwang@ciac.ac.cn gaojing@ciac.ac.cn; Laboratory for Marine Biology and Biotechnology, Qingdao National Laboratory for Marine Science and Technology Wenhai Road, Aoshanwei, Jimo Qingdao Shandong 266237 P. R. China; University of Chinese Academy of Sciences Beijing 100049 P. R. China

## Abstract

Correction for ‘Using an RNA aptamer probe for super-resolution imaging of native EGFR’ by Qiuyan Yan *et al., Nanoscale Adv.*, 2019, DOI: 10.1039/c8na00143j.

The authors regret the omission of one of the funding bodies in the Acknowledgements: Foundation of Science and Technology Department of Jilin Province (No. 20170623027TC to JJ). The corrected list of funding bodies is shown below:

This work was financially supported by the National Key R&D Program of China (No. 2017YFA0505300 to HW), NSFC (No. 21727816, 21525314, and 21721003 to HW; No. 21703231 to JG; No. 21503213 to MC; No. 31330082 to JJ), and Foundation of Science and Technology Department of Jilin Province (No. 20170623027TC to JJ).

The Royal Society of Chemistry apologises for these errors and any consequent inconvenience to authors and readers.

## Supplementary Material

